# Female sexual function following a novel transobturator sling procedure without paraurethral dissection (modified-TOT)

**DOI:** 10.1590/S1677-5538.IBJU.2016.0270

**Published:** 2017

**Authors:** Burak Arslan, Ozkan Onuk, Ali Eroglu, Tugrul Cem Gezmis, Memduh Aydın

**Affiliations:** 1 Department of Urology, Istanbul Taksim Training and Research Hospital, Turkey

**Keywords:** Urinary Incontinence, Stress, Suburethral Slings, Dyspareunia

## Abstract

**Purpose:**

To determine whether there is a difference in sexual function after modified and classical TOT procedures.

**Materials and Methods:**

Of the 80 sexually active women with SUI, 36 underwent an original outside-in TOT as described by Delorme, and 44 underwent modified TOT procedure, between 2011 and 2015. The severity of incontinence and sexual function were evaluated using International Consultation on Incontinence Questionnaire-Short Form (ICIQ-SF) and Female Sexual Function Index (FSFI) questionnaires preoperatively and 3 months after surgery.

**Results:**

The postoperative ICIQ-SF score was significantly lower than the preoperative ICIQ-SF score in both groups (p=0.004 for modified TOT and p=0.002 for classical TOT). There was no significant difference in the ICIQ-SF score reduction between the two groups (14.1±2.1 vs. 14.4±1.9; p=0.892). Complication rates according to the Clavien-Dindo classification were also similar in both groups. In both groups, difference between preoperative and postoperative FSFI scores revealed a statistically significant improvement in all domains. Comparison of postoperative 3-month FSFI scores of modified and classical TOT groups showed statistically significant differences in arousal, lubrication and orgasm domains. Desire, satisfaction, pain and total FSFI scores did not differ significantly between two groups.

**Conclusion:**

The modified TOT technique is a simple, reliable and minimal invasive procedure. The cure rate of incontinence and complication rates are the same as those of the classical TOT technique. However, due to the positive effects of minimal tissue damage on sexual arousal and orgasmic function, modified TOT has an advantage over the classical TOT.

## INTRODUCTION

Stress urinary incontinence (SUI) is extensively accepted as a social problem and defined as the involuntary leakage of urine with effort (1). Generally, causes of incontinence are hypermobility due to the loss of urethral support and lower pressure transmission to the urethra, compared to the urinary bladder (2). In the last decade, mid-urethral sling surgery has become the standard procedure for treatment of SUI in women. These procedures, including transobturator tape (TOT), tension-free vaginal tape (TVT), tension-free vaginal tape-obturator (TVT-O), and single incision sling (SIS), lead to less complications with comparable results to conventional open surgeries (3-5). To reduce the complications of retropubic sling procedure, transobturator approach was described by Delorme in 2001 (5). In spite of the high volume of reports addressing the safety and efficacy of TOT, we believe that this technique can be further improved. In support of this, Onuk et al. have described a new technique without paraurethral dissection, which is called modified TOT (mTOT).

Incontinence-related sexual dysfunction resulting from decreased libido, recurrence dermatitis induced dyspareunia and fear of coital leakage has been reported by women (6). Even though some studies have found improvement on sexual function after mid-urethral sling procedures, there are also reports on negative effects (7-9). Different sling insertion techniques and the experience of the surgeon may lead to varying outcomes. Due to the G-spot (an erogenous area in some women) located on the anterior wall of the human vagina, extensive vaginal incision and paraurethral dissection in the sling procedure can affect sexual function (10).

The aim of our study was to evaluate sexual function prospectively in women before and after surgery for SUI, and to determine whether there was a difference in sexual function between classical TOT and modified TOT procedures using Female Sexual Function Index (FSFI) questionnaire.

## MATERIALS AND METHODS

Sexually active women with stress urinary incontinence who underwent suburethral sling procedure between July 2011 and September 2015 were recruited to the prospectively planned study after receiving approval of the local ethical committee. Exclusion criteria were history of incontinence or pelvic reconstructive surgery, known psychiatric and neurological disorders, physical examination findings of above grade 1 pelvic organ prolapse. Of the 80 patients providing written informed consent, 36 underwent an original outside-in TOT as described by Delorme (5) and 44 underwent modified TOT procedure as described below. The preoperative evaluation included general history, physical examination, urine analyses, voiding dairy, urine culture and antibiogram, Marshall-Boney test, urethral Q-type test, urinary ultrasonography (to determine the amount of post voiding residue), and urodynamic evaluation (in mixed incontinence patients). The severity of urinary incontinence and its impact on quality of life (QoL) and sexual function were evaluated using International Consultation on Incontinence Questionnaire-Short Form (ICIQ-SF) and Female Sexual Function Index (FSFI) questionnaires preoperatively and 3 months after the surgery. The FSFI is a 19-item questionnaire that allow investigation of six domains; sexual desire (items 1, 2), arousal (items 3-6), lubrication (items 7-10), orgasm (items 11-13), satisfaction (items 14-16) and pain during sexual intercourse (items 17-19) (11). Total FSFI score range was 2-36.

### Surgical Procedure

**Figure 1:**
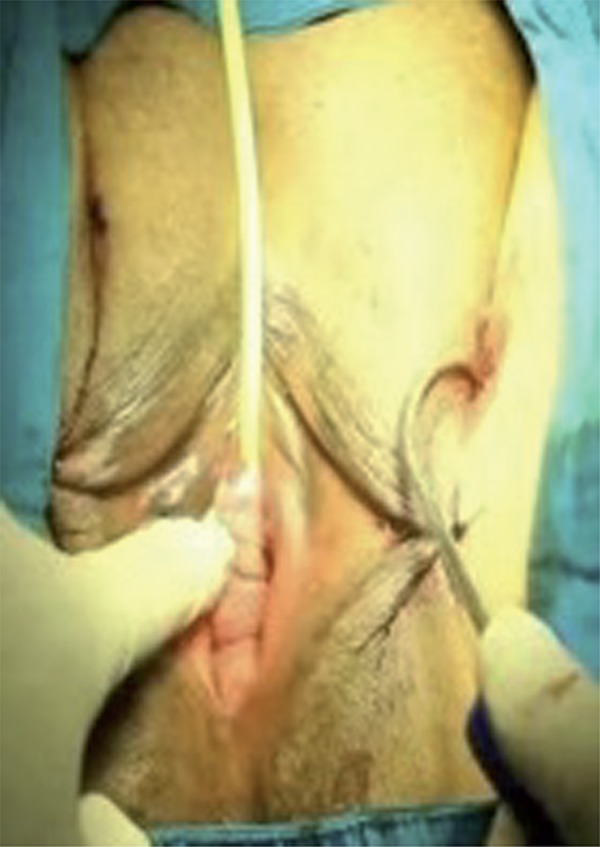
The needle is pushed until it reaches 1.5cm below the urethral meatus (left).

**Figure 2:**
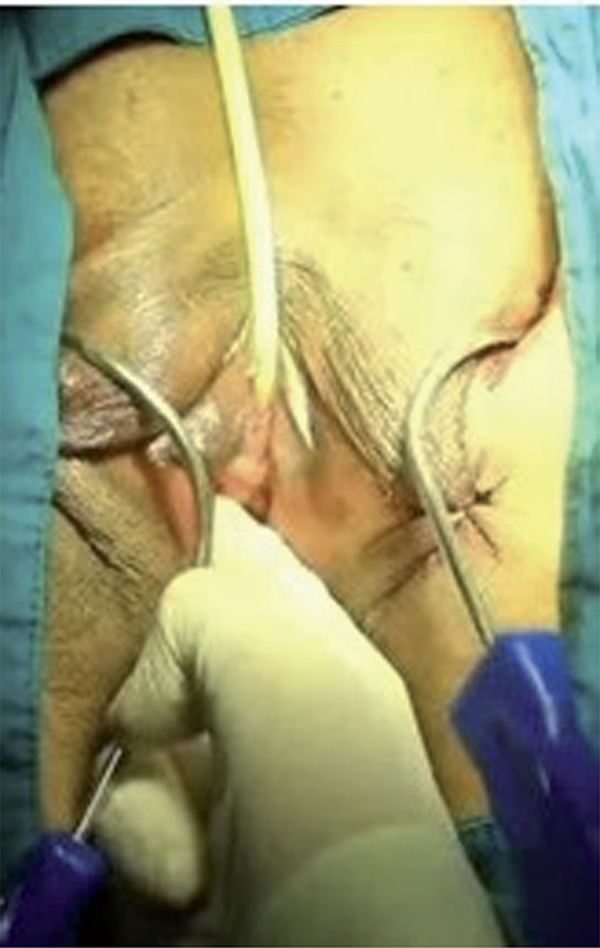
The needle is pushed until it reaches 1.5cm below the urethral meatus (right).

**Figure 3:**
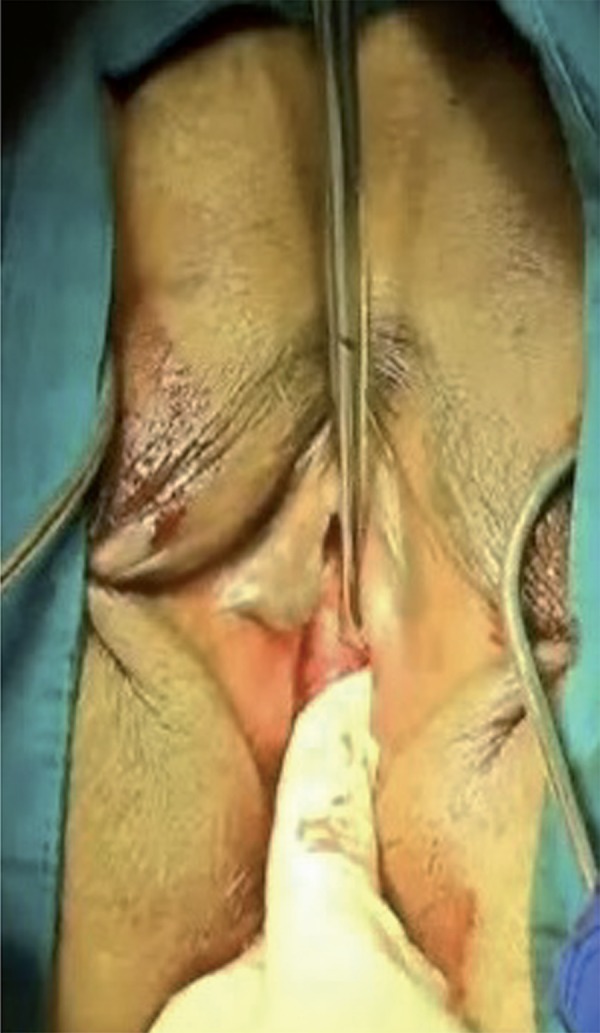
The needles meet 1.5cm below the urethral meatus.

**Figure 4:**
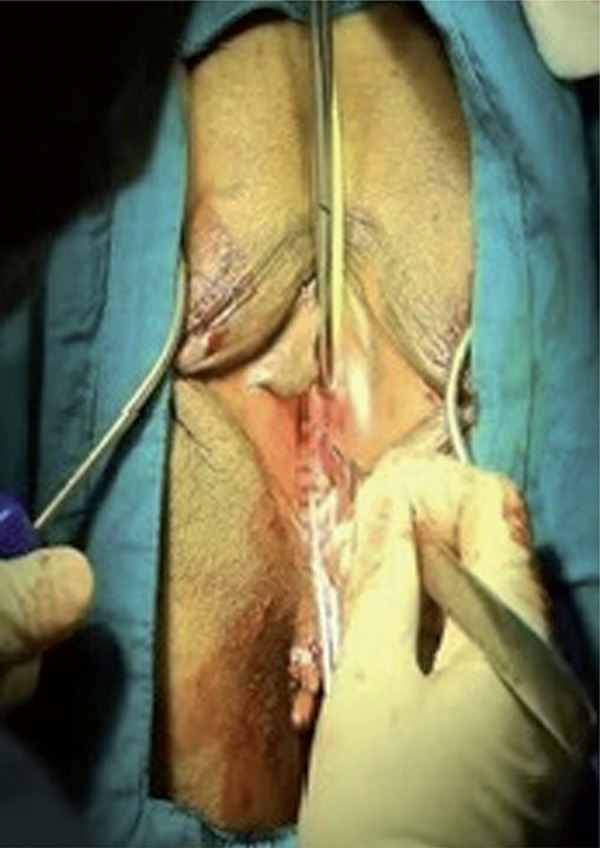
A 0.5cm cut is made, and needle points are taken outside.

The surgical area was sterilized and a Foley catheter was inserted in the lithotomy position. After the labial retraction sutures were placed, weighted vaginal speculum was inserted into the vagina. In alignment with the clitoris, 0.5cm bilateral groin incisions are made to the inferior of adductor longus muscle at the genitocrural fold level. After the helical needles were inserted the groin incisions, internal rotation through the obturator membrane was performed. Inferior ischiopubic ramus and obturator internus muscles were identified using the index finger of the opposite hand, without performing a vaginal incision. Subsequently, the needles were traversed through the obturator membrane, obturator internus muscle, periurethral endopelvic fascia and were felt under the vaginal mucosa with the index finger. During this process, anterior vaginal wall damage was not observed. In the next step, the needle was pushed until it reached 1.5cm below the urethral meatus (Figure-1). The same procedure was conducted for the opposite side as well, so that the needles met 1.5cm below the urethral meatus (Figure-2 and Figure-3), where a 0.5cm cut was made, and needle points were taken outside (Figure-4). The synthetic mesh was attached to the tips of the needles and the needles were backed out, bringing the mesh out through the level of the skin in the groin region. To arrange the tension of the mesh, a surgical clamp was placed between the mesh and the urethra. Incisions were closed with absorbable sutures.

Cystourethroscopy was performed for the first 20 patients who underwent the modified technique. However, cystourethroscopy was later removed from the routine procedure since no injury was observed. Vaginal tampons were used for the classical technique group to control bleeding, but not for the modified technique group. Foley catheters of both groups were removed on the first postoperative day, as well as the vaginal tampons. All patients were discharged on the first postoperative day. Patients were allowed to engage in sexual intercourse after the fourth postoperative week if they underwent original outside-in TOT, and tenth postoperative day if they underwent modified TOT due to better local (vaginal) conditions.

### Statistical analysis

This study hypothesized that modified TOT procedure will be superior to the classical TOT in terms of postoperative sexual function. A total of 72 patients were required to achieve 80% power with a two-sided type 1 error of 0.05. Statistical analyses were performed using SPSS 21.0 (Chicago, Illinois) and p<0.05 was considered statistically significant. Categorical variables were analyzed using Chi-square or Fisher’s exact tests, and continuous variables were analyzed using Mann-Whitney U and Kruskal-Wallis tests.

## RESULTS

Patient characteristics for modified TOT and classical TOT groups are shown in Table-1. There were no statistical differences between the two groups in terms of age, BMI, parity, menopausal, degree of education, diabetes, hypertension, smoking, frequency of intercourse and medical treatment for SUI.

**Table 1:** Comparison of baseline characteristics of patients.

	Modified TOT n=44	Classical TOT n=36	p value
Age, years	54 (32-67)	52 (36-67)	0.215
Body mass index, kg/m^2^	32 (22-41)	31 (24-40)	0.554
Parity, n	3 (0-6)	3 (2-6)	0.116
Menopausal, n (%)	31 (70.4%)	26 (72.0%)	0.862
**Degree of education**			0.941
No education	6 (13.7%)	4 (11.1%)	
≤ High school	30 (68.2%)	25 (69.4%)	
University	8 (18.1%)	7 (19.5%)	
Diabetes, n (%)	14 (31.8%)	11 (30.5%)	0.904
Hypertension, n (%)	16 (36.3%)	12 (33.3%)	0.777
Smoking, n (%)	7 (15.9%)	6 (16.6%)	0.927
Frequency of intercourse			0.882
>2/week	5 (11.3%)	5 (13.8%)	
1-2/week	28 (63.6%)	21 (58.4%)	
1-3/month	11 (25.1%)	10 (27.8%)	
Treatment (SNRI) for SUI, n (%)	17 (38.6%)	13 (36.1%)	0.816

The mean postoperative ICIQ-SF score was significantly lower than the preoperative ICIQ-SF score in both groups (16.6 vs. 2.5; p=0.004 for modified TOT and 17.5 vs. 3.1; p=0.002 for classical TOT). There was no significant difference in the ICIQ-SF score reduction between the two groups (14.1±2.1 vs. 14.4±1.9; p=0.892). Complication rates according to the Clavien-Dindo classification were also statistically similar in both groups (Table-2).

**Table 2:** Complications reported according to the Clavien-Dindo classification.

	Modified TOT	Classical TOT	p value
Grade I	2 (4.5%)	3 (8.3%)	
Dyspareunia	2	3	0.662
Grade II	6 (13.6%)	7 (19.4%)	
Inguinal pain	2	4	0.401
Urgency	2	3	0.653
Vaginal damage	2	0	0.499
Grade IIIa	1 (2.2%)	2 (5.5%)	
Urinary retention	1	2	0.585

In both groups, preoperative and postoperative FSFI scores revealed significant improvements in all domains (desire, arousal, lubrication, orgasm, satisfaction, pain and total score) (Table-3).

**Table 3:** Changes between preoperative and postoperative scores on the FSFI.

	Modified TOT			Classical TOT		

	Preoperative	Postoperative	p value	Preoperative	Postoperative	p value
Desire	3.06±0.76	3.72±0.82	<0.001	3.13±0.72	3.69±0.92	<0.001
Arousal	3.53±0.96	4.52±1.02	<0.001	3.46±0.88	4.09±0.95	<0.001
Lubrication	4.36±1.12	4.97±1.41	<0.05	4.11±1.22	4.52±1.16	<0.05
Orgasm	3.96±1.01	4.82±1.36	<0.001	4.02±1.87	4.46±0.94	<0.001
Satisfaction	4.56±0.99	5.42±1.10	<0.001	4.42±0.88	5.12±1.11	<0.05
Pain	4.12±0.78	5.21±1.34	<0.001	4.22±0.96	4.92±1.18	<0.05

**Total**	**23.59±4.12**	**28.66±5.95**	**<0.001**	**23.36±4.13**	**26.80±4.97**	**<0.001**

Comparison of postoperative FSFI scores between modified TOT and classical TOT procedures showed statistically significant differences in arousal, lubrication and orgasm domains (Table-4). Desire, satisfaction, pain and total FSFI scores did not differ significantly between two groups (Table-4).

**Table 4:** Comparison of postoperative FSFI scores for modified and classical TOT.

	Modified TOT	Classical TOT	p value
Desire	3.72±0.82	3.69±0.92	0.644
Arousal	4.52±1.02	4.09±0.95	<0.05
Lubrication	4.97±1.41	4.52±1.16	<0.05
Orgasm	4.82±1.36	4.46±0.94	<0.05
Satisfaction	5.42±1.10	5.12±1.11	0.168
Pain	5.21±1.34	4.92±1.18	0.619

**Total**	**28.66±5.95**	**26.80±4.97**	**0.328**

## DISCUSSION

Female sexual dysfunction is a major health problem associated with age, degree of education, medical and psychosocial situations, and is composed of orgasmic disorder, dyspareunia and lack of sexual desire (12). Epidemiological studies have demonstrated that approximately 40% of women have sexual problems worldwide (13). It is well known that the prevalence of sexual dysfunction in women with stress urinary incontinence is higher than healthy continent females (14). However, there are conflicting results concerning the effect of incontinence surgery on sexual function (8-10). Pastore et al. used FSFI questionnaire to evaluate sexual function in 48 women who underwent TVT-O and SIS procedures. The postoperative FSFI scores were reported to improve significantly (p<0.001) in both groups, with high rate of continence (15). Naumann et al. assessed sexual function six months after TVT and SIS surgeries and reported that, in comparison to preoperative scores, all postoperative domain scores and total FSFI score increased significantly in both surgical groups (16). Results of Simsek et al. and Abo El-Enen et al. also indicate an improvement in FSFI scores after the amelioration of incontinence using transobturator sling procedure (14, 17). In contrast, a meta-analysis of eighteen studies showed that sling surgery had negative impacts on 13.1% of patients and there was no change in symptoms for 55.5% (18). In our study, FSFI scores showed statistically significant improvements in all domains in the 3-month follow-up. We believed that sexual dysfunction in women with urinary leakage has a psychological background, and achieving continence during sexual intercourse may improve self-confidence and sexual performance.

There are numerous studies in the literature that compare postoperative sexual function of four minimal invasive surgical techniques; TOT, TVT, TVT-O and SIS. Elzevier et al. evaluated postoperative sexual complaints of 77 patients who underwent TVT-O and TOT for SUI. Jang et al. also evaluated the possible effects of two operative methods on sexual function, including retropubic route and transobturator route. FSFI scores of forty-seven patients were analyzed. In both comparative studies no difference were observed except pain during intercourse after the TOT procedure. Although the exact cause is not clear, pain disorder in the TOT group may be related to vaginal injury and narrowing, vascular or neuronal detriment (19, 20). On the other hand, the first prospective comparative study in the literature that analyzed TVT and SIS reported, no difference between postoperative pain scores for the two surgical techniques. Interestingly, a statistical difference was found for lubrication and orgasm domains, in favor of the TVT procedure (16). In another comparative study conducted by Murphy et al., 329 patients were treated with TVT or TVT-O procedures. Preoperative and postoperative sexual functions were evaluated with The Pelvic Organ Prolapse/Incontinence Impact Questionnaire (PISQ-12). The two groups did not differ significantly in terms of sexual function (21). In our study, improvement in arousal (4.52 vs. 4.09; p<0.05), lubrication (4.97 s 4.52; p<0.05) and orgasm (4.82 vs. 4.46;p<0.05) domains were significantly higher in the m-TOT group than the classical TOT group. Additionally, total FSFI score improvement was higher in women with m-TOT, but this difference was not statistically significant. (28.66±5.95 vs. 26.80±4.97; p=0.328). In the beginning of our study, we hypothesized that, in comparison to the classical TOT, a surgical approach without wide vaginal incision and paraurethral dissection would result in less postoperative pain, earlier sexual intercourse and improved sexual function. As known, extensive anterior vaginal wall incision and paraurethral dissection may impair the neurovascular tissues, induce vaginal scarring and lead to orgasmic and arousal problems (22, 23). In concordance with literature, our findings revealed that m-TOT technique has an advantage on vaginal fibrosis, preserving innervation and enhancing the orgasmic/arousal function. To resume sexual activity earlier due to the lack of introital wound tenderness is another gain of our technique.

## CONCLUSIONS

The modified TOT technique is a simple, reliable and minimal invasive procedure. The cure rate of urinary incontinence and complication rates are the same as the classical TOT technique. However, due to the positive effects of minimal tissue damage on sexual arousal and orgasmic function, modified TOT has an advantage over the classical TOT. Sexual function assessment was limited to three months after the surgery and this was the partial limitation or our study. Additional studies providing longer-term follow-up reports may improve our insight about the effects of modified TOT procedure.
